# Intercalated Disk Extracellular Nanodomain Expansion in Patients With Atrial Fibrillation

**DOI:** 10.3389/fphys.2018.00398

**Published:** 2018-05-04

**Authors:** Tristan B. Raisch, Matthew S. Yanoff, Timothy R. Larsen, Mohammed A. Farooqui, D. Ryan King, Rengasayee Veeraraghavan, Robert G. Gourdie, Joseph W. Baker, William S. Arnold, Soufian T. AlMahameed, Steven Poelzing

**Affiliations:** ^1^Virginia Tech Carilion Research Institute, Center for Heart and Regenerative Medicine, Virginia Tech, Blacksburg, VA, United States; ^2^Translational Biology, Medicine, and Health, Virginia Tech, Blacksburg, VA, United States; ^3^Virginia Tech Carilion School of Medicine, Roanoke, VA, United States; ^4^Department of Medicine, Section of Cardiology, Center for Atrial Fibrillation, Carilion Clinic, Roanoke, VA, United States; ^5^Department of Biomedical Engineering, The Ohio State University, Columbus, OH, United States; ^6^The Bob and Corrine Frick Center for Heart Failure and Arrhythmia, The Ohio State University, Columbus, OH, United States; ^7^Department of Physiology and Cell Biology, College of Medicine, The Ohio State University, Columbus, OH, United States; ^8^Department of Biomedical Engineering and Mechanics, Virginia Tech, Blacksburg, VA, United States; ^9^Department of Surgery, Carilion Clinic, Roanoke, VA, United States

**Keywords:** atrial fibrillation, gap junctions, connexin43, perinexus, human

## Abstract

**Aims:** Atrial fibrillation (AF) is the most common sustained arrhythmia. Previous evidence in animal models suggests that the gap junction (GJ) adjacent nanodomain – perinexus – is a site capable of independent intercellular communication via ephaptic transmission. Perinexal expansion is associated with slowed conduction and increased ventricular arrhythmias in animal models, but has not been studied in human tissue. The purpose of this study was to characterize the perinexus in humans and determine if perinexal expansion associates with AF.

**Methods:** Atrial appendages from 39 patients (pts) undergoing cardiac surgery were fixed for immunofluorescence and transmission electron microscopy (TEM). Intercalated disk distribution of the cardiac sodium channel Nav1.5, its β1 subunit, and connexin43 (C×43) was determined by confocal immunofluorescence. Perinexal width (Wp) from TEM was manually segmented by two blinded observers using ImageJ software.

**Results:** Nav1.5, β1, and C×43 are co-adjacent within intercalated disks of human atria, consistent with perinexal protein distributions in ventricular tissue of other species. TEM revealed that the GJ adjacent intermembrane separation in an individual perinexus does not change at distances greater than 30 nm from the GJ edge. Importantly, Wp is significantly wider in patients with a history of AF than in patients with no history of AF by approximately 3 nm, and Wp correlates with age (*R* = 0.7, *p* < 0.05).

**Conclusion:** Human atrial myocytes have voltage-gated sodium channels in a dynamic intercellular cleft adjacent to GJs that is consistent with previous descriptions of the perinexus. Further, perinexal width is greater in patients with AF undergoing cardiac surgery than in those without.

## Introduction

Atrial fibrillation (AF) is the most common cardiac arrhythmia affecting an estimated 5.2 million Americans (Colilla et al., 2013). Coordinated cardiac contraction is dependent upon organized cell-to-cell communication via the propagation of an electric signal. AF can occur secondary to disruptions in organized myocyte depolarization leading to conduction slowing and failure (Grubb and Furniss, 2001; Andrade et al., 2014).

Importantly, targetable mechanisms underlying epidemic AF are sparse. For example, a number of studies in animal models and humans have demonstrated that gap junction (GJ) remodeling is associated with abnormal atrial conduction and AF (Lübkemeier et al., 2013; Yan et al., 2013; Dhillon et al., 2014; Rothe et al., 2014). Targeting GJ remodeling remains challenging, and there is a critical need to expand knowledge and develop new therapeutic approaches by thinking outside the box. Recent studies suggest that GJs are not the only mechanism for electrical communication between cardiac myocytes. Our research suggests that ephaptic coupling, via the generation of electric fields and ion accumulation/depletion transients within restricted intercalated disk domains, can well describe a number of conduction abnormalities associated with GJ, sodium channel, and ionic modulation (Veeraraghavan et al., 2012, 2015, 2016; George et al., 2015, 2016, 2017; Entz et al., 2016).

A candidate cardiac ephapse has been identified for mediating ephaptic coupling in guinea pig and murine ventricular myocardium. This nanodomain, termed the “perinexus,” is an extracellular space in the intercalated disk directly adjacent to GJ plaques that is narrow (on the scale of 5–30 nm), can be dynamically altered, and contains a high density of the cardiac isoform of the voltage gated sodium channel (Rhett et al., 2012; Veeraraghavan et al., 2015, 2016; Veeraraghavan and Gourdie, 2016). Furthermore, we have demonstrated in animal models (George et al., 2015, 2016, 2017; Veeraraghavan et al., 2015; Entz et al., 2016; Veeraraghavan and Gourdie, 2016) that altering perinexal width is associated with altered cardiac conduction consistent with the theories of ephaptic coupling (Kucera et al., 2002; Mori et al., 2008; Lin and Keener, 2010; Hichri et al., 2018). While evidence has grown suggesting the importance of non-gap junctional coupling mediating conduction velocity in animal models, it is unknown whether the perinexus can be found in human myocardium, and more specifically the atria. The purpose of this study was to identify the perinexus in human atrial tissue and provide a robust workflow for quantifying the structure. We then investigated whether perinexal morphology differed between patients with and without known pre-existing AF.

## Materials and Methods

The study was approved by the Carilion Clinic Institutional Review Board and all subjects gave informed and written consent prior to participating in research. Procedures followed were in accordance with institutional guidelines.

Inclusion criteria for patients enrolled in the study were: Ages 18–80, undergoing elective cardiac surgery with and without MAZE procedure at Carilion Roanoke Memorial Hospital (CRMH). Exclusion criteria were: emergent surgery, recent history of endocarditis, history of infiltrative cardiac disease, history of connective tissue disease, active steroid or immunosuppressive therapy, or repeat cardiac surgical procedure.

### Tissue Collection

The day prior to the procedure, 2.5% glutaraldehyde in phosphate buffered saline (PBS) at 4°C was prepared and transferred to the cardiac surgical floor in an insulated container. During surgery, the surgeon placed tissue in the fixative immediately after resection. Since all patients undergoing a MAZE procedure had pre-operative AF, surgeons were not blinded for this study. Tissue samples were collected from the right or left atrial appendage depending on the surgeon’s method of cannulation for cardiac bypass. Sample were then brought to Virginia Tech Carilion Research Institute (VTCRI), cut into 1 mm^3^ samples, placed in newly prepared 2.5% glutaraldehyde in PBS fixative, and stored at 4°C. Samples were chosen from the periphery of the specimen to ensure fixative diffusion would be adequate to minimize *ex vivo* changes in the tissue secondary to ischemic contraction. Tissue was washed the following day, placed in PBS, and stored at 4°C prior to transport to the Virginia-Maryland College of Veterinary Medicine for transmission electron microscopy (TEM) slide preparation and imaging. 46 patients were enrolled in the study, tissue was retrieved from 39 patients, 7 patient samples were not collected as cannulation occurred outside of the atria. A total of 41 samples were collected as two patients provided samples from both the left and right atria. Three additional samples were obtained from patients without a history of AF for the purpose of immunohistochemistry.

### Immunofluorescence

Human right atrial tissue samples were fixed in paraformaldehyde (PFA), cryosectioned, and immunolabeled following previously described collection procedures (Veeraraghavan et al., 2015). Tissue samples were fixed in PFA (2%) at room temperature for 3 h, rinsed in PBS, and equilibrated sequentially in 15 and 20% solutions of sucrose at 4°C. Samples were placed into cryomolds with optimal cutting temperature (OCT) medium and frozen over liquid nitrogen. Thin sections (5 μm thickness) obtained via cryosectioning were labeled with a mouse monoclonal antibody against C×43 (Millipore MAB3067, 1:250) and a rabbit polyclonal antibody against the voltage gated sodium channel α-subunit Nav1.5 followed by goat anti-mouse Alexa Fluor 568 (1:4000) and goat anti-rabbit Alexa Fluor 647 (1:4000) secondary antibodies. A separate set of thin sections were labeled with the same mouse monoclonal antibody against C×43 and a rabbit polyclonal antibody produced by Thermo Fisher against the β1 subunit of the Nav1.5 channel, followed by the same goat anti-mouse and goat anti-rabbit (Alexa Fluor 568 and 647, respectively) secondary antibodies. Confocal imaging was performed using a TCS SP8 laser scanning confocal microscope equipped with a Plan Apochromat 63×/1.4 numerical aperture oil immersion objective and a Leica HyD hybrid detector (Leica, Buffalo Grove, IL, United States).

### Transmission Electron Microscopy

Samples were washed in PBS and processed for TEM as previously described (Veeraraghavan et al., 2015). Particular care was taken to quickly fix the samples in cooled glutaraldehyde and expose samples to identical fixation conditions in order to minimize heterogeneity in tissue fixation. The sample was sectioned onto copper grids and the sections were imaged using a JEOL JEM 1400 transmission electron microscope. The GJ was identified by locating an in-plane pentalaminar structure (Revel and Karnovsky, 1967; Huttner et al., 1973) with a continuously in-plane cell separation region extending at least approximately 150 nm from the end of the GJ that we termed the perinexus. We collected and analyzed seven images at 150,000× magnification for each of the 39 samples included in the study. Perinexal images were then analyzed by two blinded observers using ImageJ to determine perinexal width (Wp). Importantly, the Wp measurements in this study refer to the intermembrane separation adjacent to the GJ plaque as we previously reported in mice and guinea pigs (George et al., 2015; Veeraraghavan et al., 2015). In short, a perpendicular line is drawn approximately 5 nm from the edge of the GJ and beginning of the perinexus to measure inter-membrane separation, i.e., perinexal Wp. The process is repeated at 10, and 15 nm from the GJ edge, where after Wp is quantified every 15 nm.

### Statistical Analysis

Statistical analysis of the data was performed using a Chi-squared test to assess differences between expected and observed frequencies of Wp measurements. The average of all seven replicate measurements per sample was calculated and average Wp per independent sample was used to measure the Wp difference between disease-state groups, consistent with analyzing only sample averages, not replicates (Cumming et al., 2007). Differences between groups were quantified by a two-tailed Student’s *t*-test with *p* < 0.05 considered statistically significant. Additionally, a 2 × 2 Fisher’s exact test was used to analyze differences in the number of points between adjacent measurements in order to identify the terminus of the perinexal plateau. Spearman’s rank correlation was used to determine correlation between age and perinexal width. Left atrial dilation was assessed by a board certified cardiologist, and statistical difference determined by Chi-Squared analysis. All values are reported as mean ± standard deviation.

## Results

### Patient Demographics

A total of 39 patients undergoing cardiac surgery were included for quantifying perinexal separation (**Table 1**). Mean age was 67 years, 25 (64%) were male. There were 24 coronary artery bypass procedures, 22 valve procedures, 1 aortic root repair, and 10 MAZE procedures. Several patients underwent more than one procedure. Past medical history was significant for hypertension in 34 (87%) patients, diabetes mellitus in 8 (21%), chronic kidney disease (glomerular filtration rate ≤ 60 mL/min/1.73 m^2^) in 13 (33%), and left ventricular systolic dysfunction (ejection fraction < 50%) in 5 (13%). Prior to surgery 26 (67%) patients were taking beta blockers, 22 (56%) were taking angiotensin converting enzyme inhibitors or angiotensin receptor blockers, 30 (77%) were taking statins, 2 (5%) were taking amiodarone, 6 (15%) were taking calcium channel blockers, and 5 (13%) were regularly taking non-steroidal anti-inflammatory medications.

**Table 1:** Patient and procedure characteristics.

	No AF (*n* = 19)	Prior AF (*n* = 10)	New AF (*n* = 10)
**Patient characteristics:**			
Mean age (years)	65	70	69
Hypertension	16 (84%)	9 (90%)	9 (90%)
Diabetes mellitus	5 (26%)	1 (10%)	2 (20%)
Peripheral vascular disease	4 (21%)	0	1 (10%)
LVEF ≤ 45%	3 (16%)	2 (20%)	0
Chronic kidney disease (GFR < 60^∗^)	4 (21%)	4 (40%)	5 (50%)
**Current medications:**			
Beta blocker	13 (68%)	6 (60%)	7 (70%)
ACEi/ARB	10 (53%)	6 (60%)	6 (60%)
Statin	16 (84%)	4 (40%)	10 (100%)
**Surgical procedure:**			
Coronary artery bypass	15 (79%)	2 (20%)	7 (70%)
Valve procedure	7 (37%)	9 (90%)	6 (60%)
MAZE procedure	0	10 (100%)	0


**ACEi, angiotensin converting enzyme inhibitor; AF, atrial fibrillation; ARB, angiotensin receptor blocker; GFR, glomerular filtration rate; LVEF, left ventricular ejection fraction. ^∗^mL/min/1.73 m^*2*^.**

All patients with a history of AF prior to surgery underwent a modified MAZE procedure (*n* = 10: 3 persistent AF and 7 paroxysmal AF). Ten patients (25%) developed new AF in the immediate post-operative period (prior to hospital discharge). Nineteen patients (50%) did not demonstrate AF prior to or after surgery.

### Left Atrial Size and Disease State

It is well known that left atrial enlargement associates with AF recurrence and increased AF burden (Olshansky et al., 2005; Gupta et al., 2014). In our study, left atrial enlargement was quantified as normal, mild, moderate and severe based on pre-surgical echocardiographic assessment for every patient. A chi-squared analysis revealed a statistically significant relationship between disease state and degree of left atrial expansion. Specifically, LA enlargement was more severe in patients with pre-existing history of AF (*p* < 0.05, **Table 2**).

**Table 2:** Left atrial size and history of AF.

Procedure	Normal	Mild	Moderate	Severe	Row totals
AF	1	1	4	4	10^∗^
No AF	18	7	3	1	29
Column totals	19	8	7	5	39


**Left atrial dilatation is greater in patient with history of AF relative to patients with no history of AF. ^∗^p < 0.05 by chi-squared analysis.**

### Intercalated Disk Sodium Channels

**FIGURE 1:**
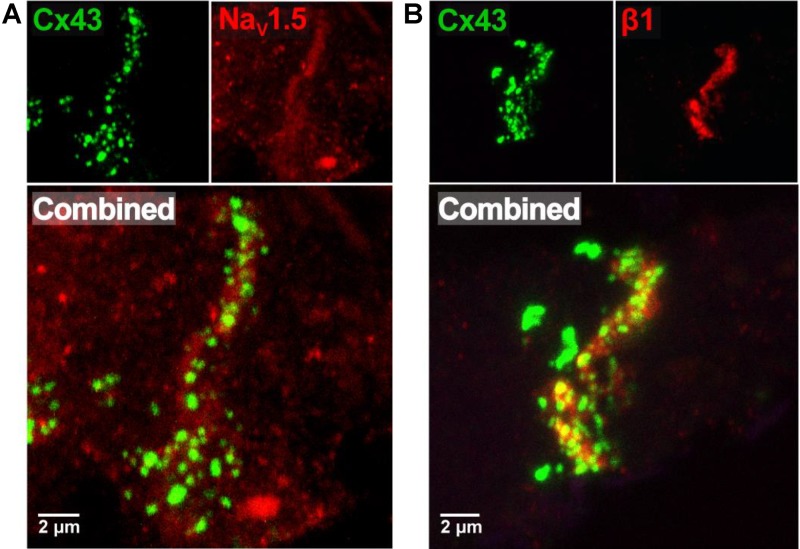
Nav1.5 and β1 enriched adjacent to connexin43 in human atria. **(A)** Representative immunofluorescent confocal image of a 5 μm-thick section of human RAA labeled for C×43 (green) and Nav1.5 (red), with both channels combined below. **(B)** Representative immunofluorescent confocal image of a similar section of human RAA labeled for C×43 (green) and the β1 auxiliary subunit of the cardiac sodium channel (red), with both channels combined below.

Previous studies suggest that dense sodium channel expression in the intercalated disk adjacent to the GJ is necessary for a non-GJ mediated form of electrical communication called ephaptic coupling. More recently, we provided evidence that the GJ-adjacent separations of membranes in the intercalated disk called the perinexus is a candidate structure for ephaptic transmission (Veeraraghavan et al., 2015). Representative confocal images from human atrial tissue in **Figure 1A** demonstrate dense immunosignals corresponding to the cardiac sodium channel α-subunit Nav1.5 (red) and connexin43 C×43 (green) at sites consistent with the intercalated disk (Veeraraghavan et al., 2015; Veeraraghavan and Gourdie, 2016), consistent with Nav1.5 enrichment within the perinexus surrounding C×43 GJs. Importantly, this GJ-adjacent Nav1.5 enrichment was consistent between samples from three different patients (data not shown). Additionally, **Figure 1B** demonstrates a similar enrichment of the β1 auxiliary Nav1.5 subunit (red) adjacent to C×43 (green).

#### Quantifying Perinexal Width

**FIGURE 2:**
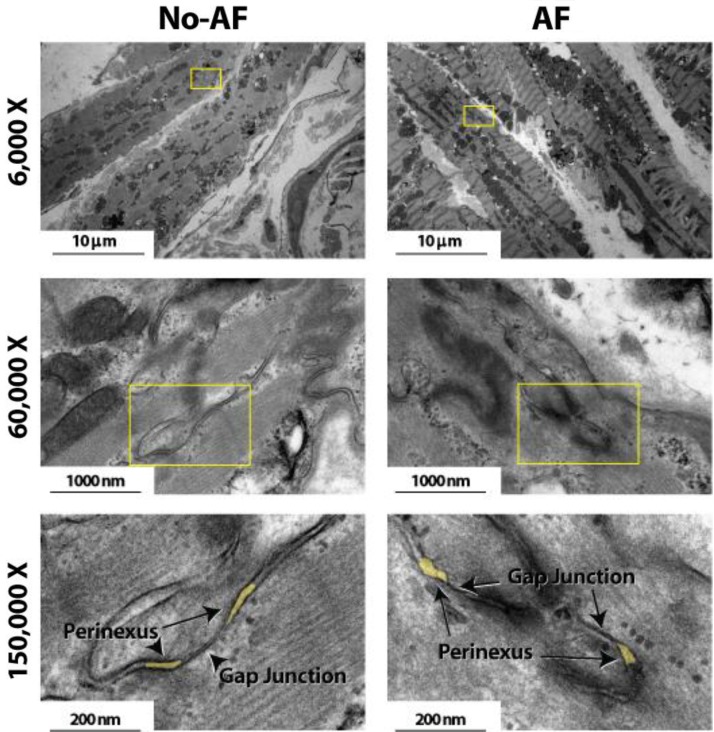
Effects of tissue fixation on tissue architecture. Transmission electron microscopy images of human atrial myocardium at 6,000, 60,000, and 150,000× magnification. Intercalated disk is the black ribbon-like structure in images. Perinexi highlighted in yellow.

**FIGURE 3:**
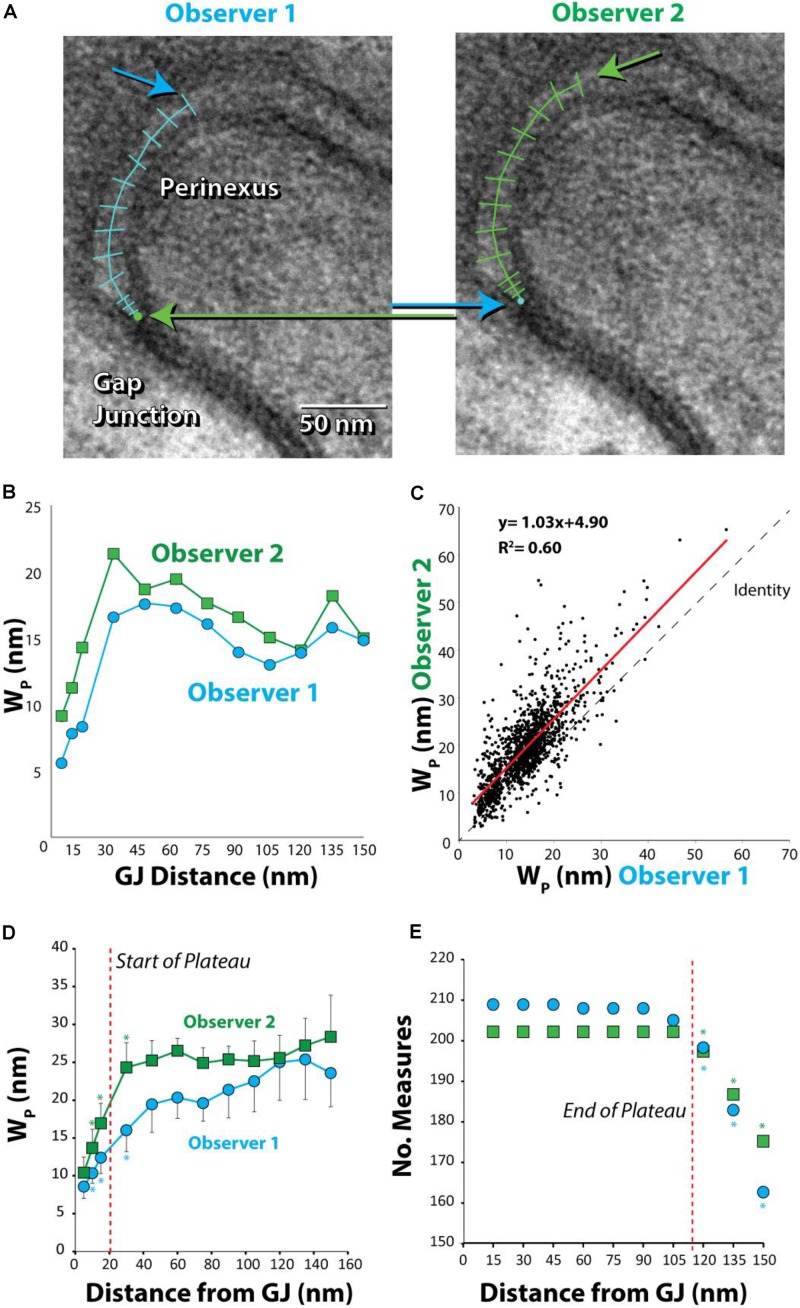
Absolute, but not relative, perinexal widths differ between observers. **(A)** Representative TEM image from human left atrial appendage measured by two different observers. Arrows indicate different starting and ending points between observers. **(B)** Perinexal width (Wp) from **(A)** are different between observers 1 and 2. **(C)** Wp correlates between observers using data from the first 20 patients (149 images) but the *y*-intercept is different. **(D)** Wp changes from the gap junction edge up to 30 nm (^∗^*p* < 0.05). **(E)** Observers collect similar numbers of Wp measurements up to 105 nm from gap junction edge. (^∗^*p* < 0.05 relative to numbers at previous distance).

Anatomical separations adjacent to GJs were quantified. Representative TEM images in **Figure 2** at 6,000 and 60,000× magnification demonstrate that tissue fixation was sufficient to minimize tissue handling artifacts, particularly at the black, ribbon-like structures in the images, which are the intercalated disks. The image of a single perinexus from an atrial appendage of a human patient is shown in **Figure 3A** at 150,000× magnification, demonstrating separation of cell membranes immediately adjacent to the GJ plaque. Linearizing perinexal width as a function of distance in **Figure 3B**, we find that both observers report Wp increases as a function of distance from the GJ edge.

Comparing paired measurement from the first 20 samples between observers 1 and 2 reveals that measurements are well-correlated between observers (*R*^2^ = 0.60, **Figure 3C**). Importantly, the slope (1.03 ± 0.02) is not significantly different from the line of identity, demonstrating that observers measure similar changes in perinexal width. However, the *y*-intercept is significantly different from 0 (4.90 ± 0.36) demonstrating that one observer consistently measures a perinexal width on average 4.90 nm greater than the other. Further, mean Wp at each spatial extent and standard error for two observers are plotted in **Figure 3D**. Wp at one distance was compared to the value at the preceding distance. For example, both observers report that Wp at 10 nm is statistically greater than the measurement at 5 nm (^∗^). After 30 nm from the GJ edge, there were no significant differences between Wp measurements. Thus, the beginning of the perinexal plateau was defined at 30 nm from the start of the perinexus.

In order to reduce the statistical confounder of including an unequal spatial extent for different images, we sought to define a robust end to the perinexus plateau using a 2 × 2 Fisher’s exact test for the number of measurements between adjacent spatial extents. For example, each observer measured equal numbers of points at 15 and 30 nm, as shown in **Figure 3E**. Importantly, statistical differences between the number of measurements at adjacent distances occurred beyond 105 nm from the GJ for both observers. Therefore 105 nm was considered the farthest spatial extent for reliably estimating the perinexal plateau as denoted by the red vertical line. As a result, we defined mean Wp for each image using measurements between 30 and 105 nm from the edge of the GJ plaque.

#### Perinexal Width and Surgical Procedure

**FIGURE 4:**
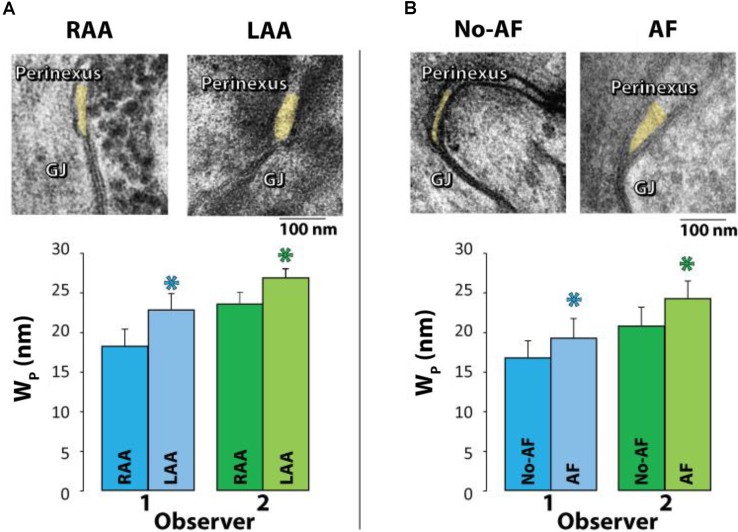
Perinexal width correlates with pre-existing atrial fibrillation. **(A)** Representative TEM image from the Right (RAA, *n* = 8) and Left (LAA, *n* = 4) atrial appendages from the same patient (top). Wp is wider in LAA relative to RAA independent of observer (bottom). **(B)** Representative TEM images from the RAA of a patient with a history of AF (MAZE procedure, *n* = 10) and without a history of AF (*n* = 29) (top). Both blinded observers found in 39 patients that mean Wp between 30 and 105 nm is significantly larger in patients with pre-existing AF relative to those without (^∗^*p* < 0.05, bottom).

Samples were collected from only the right atrial appendage (RAA) in patients without a history of AF, but a combination of left atrial appendages (LAA) and/or RAA were collected from patients with a history of AF. Representative TEM images are provided in **Figure 4A** (top panel) of GJs and perinexal regions from the same patient with a history of AF. Mean Wp for each patient was calculated from every Wp measured between 30 and 105 nm for 7 images per patient, yielding a single mean Wp per patient shown in **Figure 4A** (bottom panel) and **Table 3**. Summary data from observers 1 and 2 demonstrates that Wp is wider in LAA relative to RAA in patients with a history of AF.

**Table 3:** Summary data of perinexal width.

	Perinexal width, Wp (nm)					
	
Observer	RAA-AF (*n* = 8)	LAA-AF (*n* = 4)	No-AF (*n* = 29)	AF (*n* = 12)	No POAF (*n* = 19)	POAF (*n* = 10)
1	18.0 ± 1.2	22.6 ± 2.1^∗^	16.9 ± 2.1	19.5 ± 2.5^∗^	16.5 ± 2.2	17.4 ± 2.3
2	23.3 ± 1.5	26.7 ± 2.1^∗^	20.7 ± 2.4	24.4 ± 2.2^∗^	20.8 ± 2.5	20.7 ± 2.4


*LAA, left atrial appendages; RAA, right atrial appendages; POAF, post-operative atrial fibrillation ; AF, pre-existing AF. Statistical significance ^∗^ for p < 0.05 for individual observers determined by Student’s t-test.*

Next, we tested if mean Wp is greater in patients with pre-existing AF relative to those patients without a documented case of pre-existing AF. Representative TEM images are provided in **Figure 4A** (top panel) of GJs and perinexal regions from an RAA of a patient with a history of AF and a patient without a history of AF undergoing a cardiac procedure. To reduce the confounder of the four AF patients with both RAA and LAA samples, the LAA samples were excluded from analysis. Importantly, summary data from 39 patients (7 images per patient) in **Figure 4B** (bottom panel) and **Table 3** demonstrates that both observers found patients with preoperative AF had significantly wider perinexi than non-AF patients undergoing cardiac surgery. Additional TEM images from non-AF and AF patients are provided in Supplementary Figures S1, S2.

#### Perinexal Width, Post-operative AF, P-Wave Duration, and Age

In contrast to the relationship between pre-existing AF and Wp, we did not find a significant relationship between Wp and whether a patient *without* pre-existing AF developed post-operative AF (*n* = 10 patients) prior to discharge (**Table 3**). Furthermore, there was no significant relationship between p-wave duration and Wp. However, this is expected as p-wave duration and AF are not tightly correlated due to factors such as the diverse etiologies leading to AF.

**FIGURE 5:**
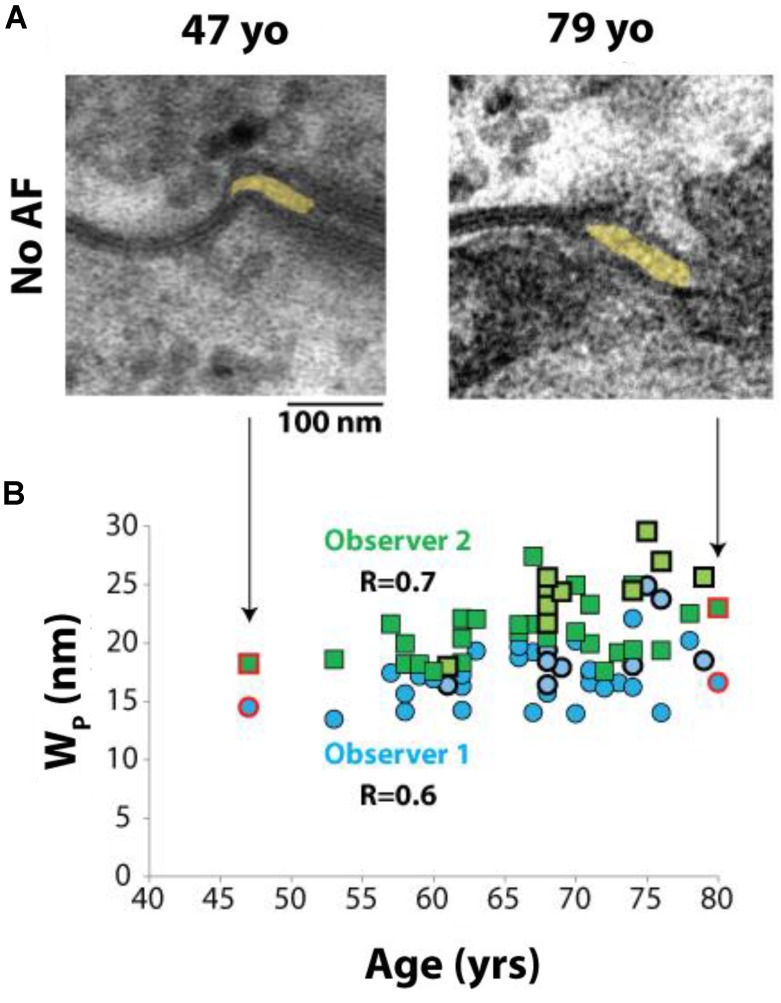
Perinexal width correlates with age. **(A)** Representative TEM image from two patients without a history of atrial fibrillation, ages 47 and 79 years old (yo). **(B)** All blinded observers found a correlation by Spearman’s Rank correlation for all patient samples analyzed (Observer 1, *R* = 0.6. Observer 2, *R* = 0.7). Dark blue circles and dark green squares represent non-AF Wp values obtained by Observers 1 and 2, respectively, while bold-outlined light blue circles and bold-outlined light green squares represent AF Wp values obtained by Observers 1 and 2, respectively. Red circles and squares indicate samples represented by TEM images in **(A)**.

Interestingly, a relationship between Wp and age was observed in a *post hoc* analysis. Representative images from 49 and 79 year old patients without a history of AF can be seen in **Figure 5A**. Spearman’s rank correlation was used to test the relationship between age and Wp in all 39 patients. Mean Wp was 19.7 nm (range 16–27 nm), mean age was 67 years (range 47–80 years). Importantly, Wp positively correlated with age, (**Figure 5B**), both in patients with and without AF.

## Discussion

This is the first study to identify and assess the anatomic features of the perinexus in human cardiac tissue. The study suggests that this sodium channel-rich separation of apposed membranes immediately adjacent to GJs is a conserved nanodomain in atrial and ventricular tissue that is species-independent: murine, (George et al., 2015) guinea pig, (Veeraraghavan et al., 2015) or human. Further, this study is the first to demonstrate that mean perinexal width is a correlate of a human disease and age.

### Na_V_1.5, and β1 Enriched Around C×43

We previously demonstrated that Nav1.5 is enriched immediately adjacent to C×43 in confocal images of guinea pig, murine ventricles, as well as neonatal rat ventricular myocytes, indicating the GJ perinexus could function as a cardiac ephapse (Rhett et al., 2011; George et al., 2015; Veeraraghavan et al., 2015; Veeraraghavan and Gourdie, 2016). Here, we demonstrate a similar enrichment in human atrial tissue where immunosignals corresponding to Nav1.5 and its auxiliary subunit, β1 (SCN1B), were enriched immediately proximal to punctae of dense C×43 immunosignal consistent with GJ plaques. In light of our previous results, these data are consistent with perinexal enrichment of Nav1.5 and β1 in human atria, indicating the presence of functional sodium channels at these perinexal sites (Calhoun and Isom, 2014; Namadurai et al., 2015).

#### Subjective Measure of Wp

This is the first study to assess perinexal width in human hearts, and systematically measure variability of measurement between multiple observers in any tissue. Previously, we reported that perinexal separation in guinea pig ventricular myocardium is approximately 12 nm (Veeraraghavan et al., 2015). We later found with different observers that perinexal separation can vary between 10 and 25 nm depending on the *ex vivo* perfusate used to sustain the heart through studies (George et al., 2015, 2016, 2017; Entz et al., 2016; Greer-Short et al., 2017).

Interestingly, previous studies quantified mean Wp at distances greater than 30 nm from the edge of the GJ, and this is consistent with the 30 nm found here. Further, the mean Wp measured in human atrial tissue in patients without pre-operative AF was consistent with results from murine and guinea pig ventricles under control conditions, suggesting that the anatomical structure is conserved across species. Altogether, the present and previous studies suggest that some differences between Wp measurements between studies may be observer-dependent. Importantly, we demonstrate here that statistically significant, disease state-dependent differences in Wp were observer independent, despite absolute differences found between observers. These results suggest that Wp could serve as a useful biomarker for cardiac disease. However, caution should be exercised when comparing absolute perinexal values between different studies and different observers.

#### Extracellular Expansion and AF

While this is the first study to correlate extracellular nanodomain expansion with AF, our findings are consistent with a recent study that found an increase in cardiac extracellular volume determined by MRI is a strong, independent predictor of recurrent AF following pulmonary vein isolation (Neilan et al., 2014). While these data reveal intriguing correlations, the approximate three orders of magnitude difference in scale between the domains quantified by MRI and TEM make it unlikely that perinexal expansion significantly contributes to the MRI signal. Additionally, a causal relationship remains to be established since both extracellular and perinexal volumes can be modulated by multiple factors which also produce other effects not considered here. For example, increased extracellular volume can be attributed to increased vascular permeability triggered by inflammatory cytokines. These inflammatory cytokines can alter cardiac electrophysiology by modulating functional protein expression in myocytes (Hansen et al., 1994; Fernandez-Cobo et al., 1999; Fernández-Velasco et al., 2007; Chappell et al., 2009; Grandy and Fiset, 2009; Guillouet et al., 2011). Furthermore, we have demonstrated that perinexal width can be modulated by extracellular free calcium concentrations and osmotic stress, both of which could also alter cardiac electrophysiology via second messenger pathways and stretch-activated channels (Aguettaz et al., 2017). While the relative changes to perinexal width are small, they are similar to what has been previously reported experimentally and what has been suggested mathematically as being electrophysiologically relevant (Mori et al., 2008). Further investigation will be required to determine whether perinexal expansion is a causal factor for AF, or a sequela of AF.

#### Extracellular Expansion and Arrhythmia Mechanisms

Atrial fibrillation incidence increases dramatically with age, (Feinberg et al., 1995) and this study demonstrates that Wp also increases with age in patients with and without AF. Our recent studies suggest that patients with wide perinexi may be at higher risk for arrhythmogenic conduction slowing (Veeraraghavan et al., 2015). Perinexal expansion likely slows conduction by modulating ephaptic cell-to-cell coupling (George et al., 2015; Veeraraghavan et al., 2015; Veeraraghavan and Gourdie, 2016). Theoretical studies of ephaptic coupling identify at least two structural requirements for ephaptic coupling: cellular separation must be relatively narrow, with intercellular cleft widths on the order of 5–30 nanometers, (Kucera et al., 2002) and abutting membranes should densely express highly conductive ion channels. This is the first study to demonstrate that atrial myocardium meets both of these criteria. Further, increased perinexal separation in AF patients, and as we have reported in animal experiments, likely reduces this form of electrical communication even if GJ functional expression is nominal. Lastly, GJ remodeling, which has been extensively reported in persistent or paroxysmal AF, is also expected to modulate ephaptic-mediated conduction disturbances as we previously suggested but this requires additional investigation. Another critical extracellular factor to consider is fibrosis, which has been strongly associated with AF (Levy, 1997; Kostin et al., 2002; Boldt et al., 2004; Xu et al., 2004; Chimenti et al., 2010; Csepe et al., 2017). However, we still do not understand the exact mechanism by which fibrosis can lead to AF and whether fibrosis *per se* and not other associated derangement, is the critical culprit for generation of AF. Our study, while not proving a causal relationship between Wp and AF, similarly shows an association between extracellular spaces and disrupted conduction. Our data suggests the perinexus could be yet another extracellular factor contributing to an arrhythmogenic substrate.

#### Limitations

Perinexal separations were obtained from 2-dimensional electron micrographs, which cannot account for the 3-dimensional structure of the perinexus. As a result, bias can be introduced into perinexal quantification. For example, images may be selected based on non-formalized criteria such as the quality of contrast, or the extent of visible membranes from the edge of a GJ. These limitations may be accounted for by increasing both the number of perinexi measured per heart as well as by increasing the total number of hearts sampled. Additionally, the absolute values of perinexal width should be interpreted cautiously, not just because an observer may segment an image differently, but also because data obtained from an *ex vivo* glutaraldehyde-fixed preparation may not accurately reflect the *in vivo* anatomy. Taken together, measurements of the perinexus are not an exact estimate of perinexal separation *in vivo.* Additional consideration should be given to the fact that an atrial appendage will not represent biological heterogeneity expected in whole atria. Furthermore, only a few LAA samples were studied, and the results may not be generalized to other patient population and all findings are hypothesis generating and mandate further validation using larger cohort.

We additionally explored the association between the perinexus and AF while considering the confounding effects of age and atrial size using a multi-logistic regression. The incidence of AF, categorical data, was defined as the dependent variable with independent variables perinexal width, left atrial size and age. While LA size was found to have the only significant relationship with AF incidence (*p* < 0.05), a low *R*-squared (0.3), high residual standard error (49% of the total estimate) and relatively low *F*-statistic (6.3) indicate the multi-logistic regression poorly fits our data, possibly due to our relatively small sample size for three independent variables. As AF is a complex disease and this study merely scratches the surface of mechanistic insight, it is worth future investigation to more fully understand the interaction between aging and structural changes regarding AF onset and perpetuation.

## Conclusion

An extracellular nanodomain adjacent to TEM-identified GJ plaques in humans, the perinexus, has dense sodium channel expression and anatomical features consistent with the perinexus in guinea pig and murine hearts. This nanodomain is wider in patients with history of AF undergoing cardiac surgery with and without MAZE procedure compared to those without a history of AF. Therefore, future studies may consider extracellular nanodomain remodeling as an important correlate of cardiac arrhythmias, and therapies designed to return these spaces to a more normal geometry may prevent arrhythmias.

## Author Contributions

All authors were responsible for writing, reviewing, and approving the manuscript. SP and SA were responsible for the overall direction of the project. TR and MY were responsible for the tissue processing and image quantification. TL was responsible for the image quantification. MF was responsible for the patient data analytics and chart review. RV and RG were responsible for the confocal immunofluorescence portion of the project. JB and WA were responsible for designing the tissue collection portion of the project.

## Conflict of Interest Statement

The authors declare that the research was conducted in the absence of any commercial or financial relationships that could be construed as a potential conflict of interest. The reviewer DC and handling Editor declared their shared affiliation.
