# Supplementary material for: Intercalated Disk Extracellular Nanodomain Expansion in Patients With Atrial Fibrillation
Source: Front Physiol. 2018 May 4;9:398. doi: 10.3389/fphys.2018.00398 (PMC5945828; doi:10.3389/fphys.2018.00398)
Supplement: Supplementary file 1 [file Image_1.PDF]

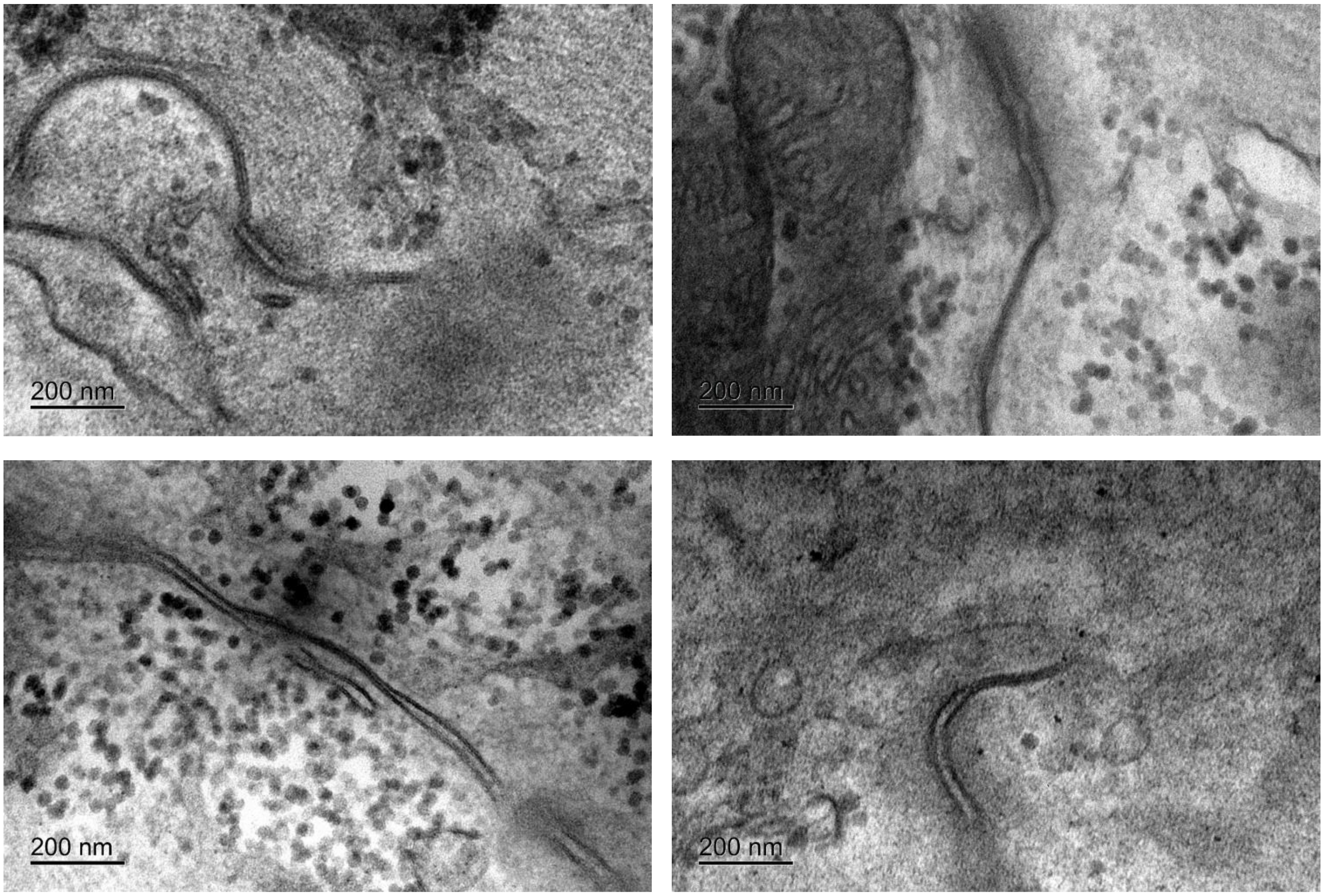

Figure S1. Representative TEM images of gap junctions and perinexi from 4 Non-AF patients

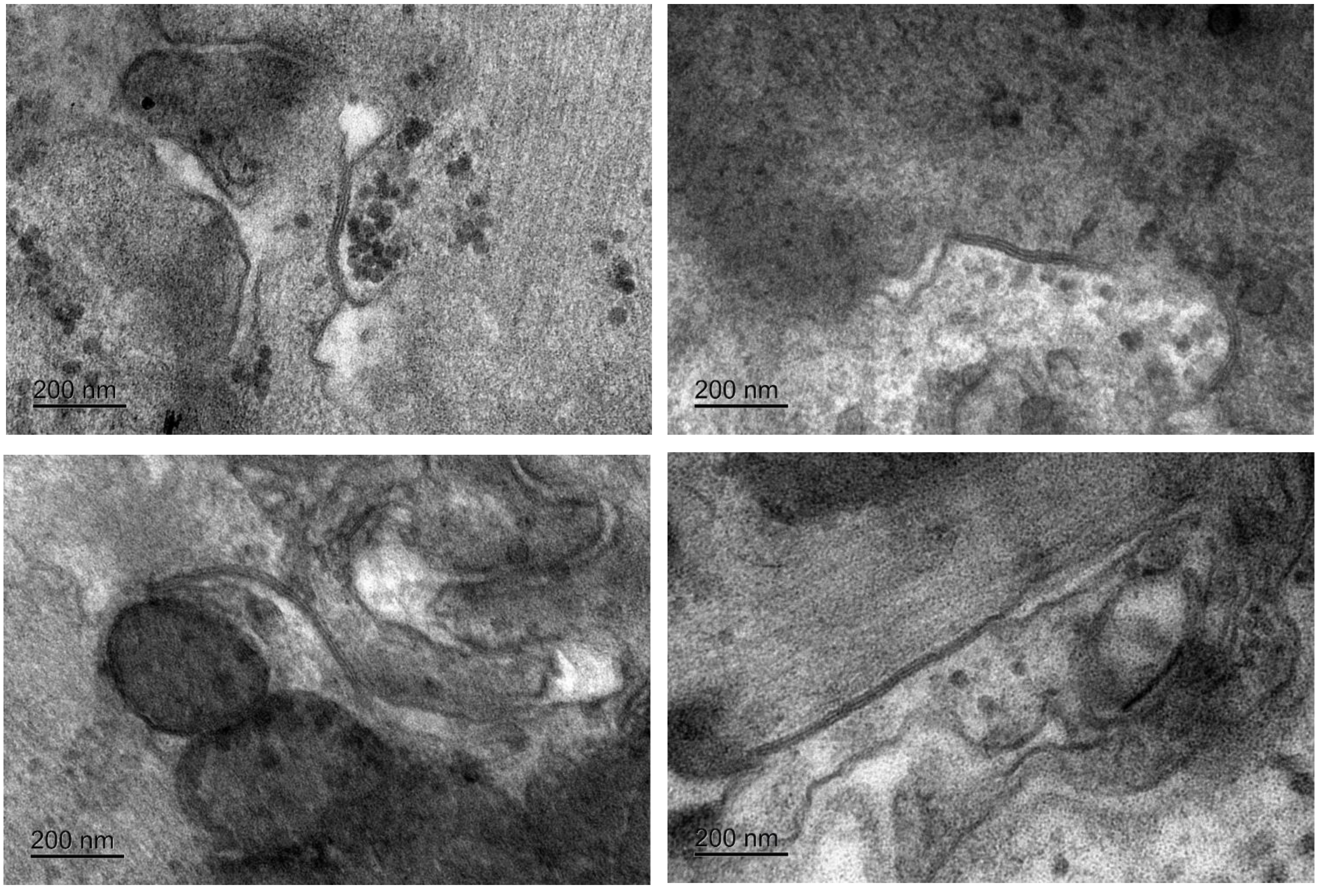

Figure S2. Representative TEM images of gap junctions and perinexi from 4 AF patients
